# Effect of non-invasive brain stimulation on cognitive function and activities of daily living in patients with carbon monoxide poisoning: a systematic review and meta-analysis

**DOI:** 10.3389/fneur.2025.1585901

**Published:** 2025-08-12

**Authors:** Hui Ding, Chen Wei, Jiwei Chen, Fen Yu, Xing Wang, Yuanwen Zhang

**Affiliations:** ^1^School of Physical Education, Shanghai Normal University, Shanghai, China; ^2^School of Physical Education, Shenyang Normal University, Shenyang, China; ^3^School of Physical Education, Shanghai University of Sport, Shanghai, China

**Keywords:** non-invasive brain stimulation, carbon monoxide poisoning, cognitive function, activities of daily living, systematic review, meta-analysis

## Abstract

**Objective:**

This study aimed to evaluate the effectiveness of non-invasive brain stimulation (NIBS) in improving cognitive function and activities of daily living (ADL) in patients with delayed encephalopathy after acute carbon monoxide poisoning (DEACMP). It also sought to explore the moderating effects of age, intervention duration, latency period, and stimulation site.

**Methods:**

A systematic search of seven databases was conducted to identify randomized controlled trials (RCTs) published up to August 2024. Meta-analyses and publication bias assessments were performed using RevMan 5.4.1 and Stata 17.0. Methodological quality was evaluated with the PEDro scale, and the certainty of evidence was assessed using GRADEpro.

**Results:**

A total of eight RCTs involving 607 participants were included. The pooled results indicated that NIBS significantly improved cognitive function (standardized mean difference (SMD = 1.03, *p* < 0.00001) and ADL (SMD = 1.77, *p* < 0.00001). The subgroup analyses showed greater cognitive improvements in patients aged below 50 years, with intervention durations of ≤20 days and stimulation applied at the yin–yang poles. In contrast, improvements in daily activities were more pronounced in patients aged over 50 years under similar intervention conditions.

**Discussion:**

The included studies were of moderate-to-high quality (mean PEDro score = 6.3). The major limitations included inadequate blinding and incomplete allocation concealment. The heterogeneity observed was mainly attributable to patient age, stimulation site, and intervention duration. No significant publication bias was detected. Overall, NIBS demonstrated moderate-quality evidence in enhancing cognitive function and daily activity performance, with individual characteristics moderating its effects.

**Systematic review registration:**

https://www.crd.york.ac.uk/prospero/, identifier CRD42024598815.

## 1 Introduction

Delayed encephalopathy after acute carbon monoxide poisoning (DEACMP) represents the most common and severe neurological complication associated with CO exposure (1, 2). Its incidence rate ranges from 20 to 40%, with mortality rates reaching up to 31% (3, 4). Affected individuals frequently develop a spectrum of delayed neuropsychiatric sequelae, including cognitive deficits, motor dysfunction, and psychiatric symptoms (5), all of which are linked to poor prognosis and elevated disability rates (6). Among these, cognitive impairment is the most prominent clinical manifestation and is closely associated with diminished capacity in activities of daily living (ADL) (7, 8).

Treatment outcomes for DEACMP vary widely, and many patients fail to achieve full neurological recovery. Approximately one-quarter of patients develop irreversible deficits (9), leading to a substantial socioeconomic burden (10). The current standard of care—pharmacological treatment combined with hyperbaric oxygen (HBO) therapy—faces significant limitations, such as high cost, limited indications, and adverse effects. These challenges underscore the urgent need for accessible and effective rehabilitation strategies.

Non-invasive brain stimulation (NIBS) has gained increasing attention due to its safety, minimal side effects, and favorable tolerability profile (11). Preliminary findings suggest the potential benefits of NIBS in DEACMP rehabilitation (1); however, inconsistencies remain. Some studies have utilized event-related potentials (ERPs), particularly the P300 component, to quantify cognitive improvement. While reductions in P300 latency are generally considered indicative of cognitive recovery, recent investigations report inconsistent changes in ERP-P300 latency in response to NIBS among patients with DEACMP (12), casting doubt on the robustness of its effects.

Against this backdrop, the present study conducted a systematic review and meta-analysis to assess the efficacy of NIBS in improving cognitive function and ADLs in patients with DEACMP. In addition, we examined whether outcomes varied as a function of patient age, onset latency, intervention duration, and stimulation site. The goal is to offer evidence-based guidance for the clinical application of NIBS in neurorehabilitation and to inform more individualized treatment protocols.

## 2 Data and methods

This study followed the PRISMA guidelines (13) for the selection and use of research methods and is registered with the Prospective International Registry for Systematic Evaluation (PROSPERO) (No. CRD42024598815).

### 2.1 Research framework

This study was conducted under the theoretical guidance of the *International Classification of Functioning, Disability and Health* (ICF) framework (14). It investigated the effects of (NIBS) on cognitive function and ADL in patients with (DEACMP). Factors including patient age, onset latency, intervention duration, and stimulation site were analyzed to determine their moderating effects. The PICOS framework adopted for this review is summarized in Table 1.

**Table 1:** PICOS framework for non-invasive brain stimulation interventions targeting cognitive function in patients with delayed encephalopathy after carbon monoxide poisoning.

Population	Intervention	Comparison	Outcome	Study design
Patients with delayed encephalopathy after carbon monoxide poisoning (first episode, age ≥18)	Rehabilitation therapists Hospital-based interventions Brain stimulation techniques: Magnetic stimulation Electrical stimulation EEG-based synchronous bioelectric stimulation	… Brain stimulation group versus control group … Comparison of stimulation durations … Comparison of stimulation target points …Stratified analysis by age and disease latency	Cognitive function (ADL)	(RCT)

### 2.2 Search strategy

A systematic search was independently conducted by two researchers across seven databases: Embase, Web of Science, PubMed, The Cochrane Library, Wanfang, VIP, and China National Knowledge Infrastructure (CNKI). The search included randomized controlled trials (RCTs) examining the efficacy of NIBS on cognitive function and ADL in patients with DEACMP, covering the period from database inception to August 2024. Manual reference checks were performed to identify additional eligible studies. The detailed search strategy is presented in Table 2.

**Table 2:** Literature search strategy.

Comprehensive database	Search step
PubMed and the Cochrane Library	#1 “Carbon monoxide poisoning delayed encephalopathy” [Mesh] OR “carbon monoxide” [Title/Abstract] OR “Carbon monoxide poisoning” [Title/Abstract] OR “Illuminating Gas Poisoning” [Title/Abstract] #2 “Anodal Stimulation Transcranial Direct Current Stimulation” [Mesh] OR tDCS [Title/Abstract] OR “Transcranial direct current stimulation” [Title/Abstract] OR “Transcranial alternating current stimulation” [Title/Abstract] OR “repetitive transcranial magnetic stimulation” [Title/Abstract] #3 “Cognitions” [Mesh] OR “Cognitive function” [Title/Abstract] OR “Cognitive Functions” [Title/Abstract] #4 Randomized controlled trial [Publication Type] OR “Randomized” [Title/Abstract] OR “controlled” [Title/Abstract] OR “Trial” [Title/Abstract] #5 #1 AND #2 AND #3 AND #4
Web of science	#1 TS = (“Carbon monoxide poisoning delayed encephalopathy” OR “carbon monoxide” OR “Carbon monoxide poisoning” OR “Illuminating Gas Poisoning”) #2 TS = (“Anodal Stimulation Transcranial Direct Current Stimulation” OR “Transcranial direct current stimulation” OR “repetitive transcranial magnetic stimulation”) #3 TS = (“Cognitions” OR “Cognitive function” OR “Cognitive Functions”) #4 TS = (“Randomized controlled trial” OR “Randomized” OR “Controlled” OR “Trial”) #5 #1 AND #2 AND #3 AND #4
Embase	#1 “Carbon monoxide poisoning delayed encephalopathy” [exp] OR “carbon monoxide” [ab,ti] OR “Carbon monoxide poisoning” [ab,ti] OR “Illuminating Gas Poisoning” [ab,ti] #2 “Anodal Stimulation Transcranial Direct Current Stimulation” [exp] OR “Transcranial direct current stimulation” [ab,ti] OR “repetitive transcranial magnetic stimulation” [ab,ti] #3 “Cognitions” [exp] OR “Cognitive function” [ab,ti] OR “Cognitive Functions” [ab,ti] #4 “Randomized controlled trial” [exp] OR “Randomized” [ab,ti] OR “Controlled” [ab,ti] OR “Trial” [ab,ti] #5 #1 AND #2 AND #3 AND #4
CNKI	主题 = (一氧化碳+一氧化碳中毒+一氧化碳中毒迟发性脑病) AND主题 = (电刺激 + 经颅直流电刺激+经颅交流电刺激+磁刺激 + 非侵入脑刺激)AND主题 = (认知功能+日常生活活动能力)
Wanfang and VIP	主题 = (一氧化碳OR一氧化碳中毒OR一氧化碳中毒迟发性脑病) AND主题 = (电刺激OR经颅直流电刺激OR经颅交流电刺激0R磁刺激0R非侵入脑刺激)AND主题 = (认知功能+日常生活活动能力)

### 2.3 Inclusion and exclusion criteria

#### 2.3.1 Inclusion criteria

Studies were included if they met the following criteria: (1) Diagnosis of DEACMP based on the 2021 Chinese Expert Consensus on the Diagnosis and Treatment of Delayed Encephalopathy After Carbon Monoxide Poisoning (10); (2) patients with a confirmed history of acute CO poisoning, aged ≥18 years; (3) interventions involving NIBS (electrical or magnetic stimulation) either alone or in combination with conventional treatment, with a minimum duration of 2 weeks; (4) control group receiving standard rehabilitation therapy; (5) primary outcomes including cognitive assessments using the mini-mental state examination (MMSE) or Montreal Cognitive Assessment (MoCA), and secondary outcomes involving activities of daily living (ADL) measures, including the Functional Independence Measure (FIM), modified Barthel Index (BI), or ADL scale; and (6) study design classified as a RCT.

#### 2.3.2 Exclusion criteria

Studies were excluded if they: (1) Were not published in Chinese or English; (2) were duplicate publications; (3) had incomplete or non-extractable data; (4) did not meet the intervention or outcome measure criteria; and (5) lacked full-text availability.

### 2.4 Data extraction and coding

All retrieved references were imported into EndNote for duplicate removal. Furthermore, two researchers independently screened the literature and extracted data using a double-blind procedure. Discrepancies were resolved through discussion with a third researcher. Data extracted included the following: the first author’s name, year and country of publication, participant demographics (age, sex), intervention details (duration, stimulation site), and outcome indicators.

Categorization of moderator variables was based on previous studies as follows: Age: ≤50 versus >50 years (15); intervention duration: ≤20 versus 21–30 days (16); stimulation site: bilateral (anodal–cathodal), left dorsolateral prefrontal cortex (DLPFC), or other locations (17); and latency period: <20 versus ≥20 days (18).

### 2.5 Quality assessment

The methodological quality of the included studies was assessed using the PEDro scale (19). Each item met was awarded one point, with a maximum possible score of 10. Scores were interpreted as follows: <4 (low quality), 4–5 (moderate quality), 6–8 (good quality), and 9–10 (high quality). Only studies rated as moderate quality or above were included in the final analysis.

The GRADEpro system was used to assess the certainty of evidence for each outcome, with ratings graded as high, moderate, low, or very low. In addition, two researchers independently performed the grading, and any discrepancies were resolved through consensus with a third reviewer.

### 2.6 Data analysis

Meta-analyses were performed using RevMan 5.4.1. All outcome variables were continuous and expressed as the mean and standard deviation (SD) of pre- and post-intervention changes. For outcomes with consistent units, the mean difference (MD) was used; for those with heterogeneous measurement scales, the standardized mean difference (SMD) was applied. Heterogeneity was assessed using the *I*^2^ statistic and *p*-values. A *p*-value of < 0.1 and *I*^2^ > 50% suggested the presence of considerable heterogeneity across the studies, thereby necessitating the application of a random effects model. Conversely, a *p*-value of ≥0.1 and *I*^2^ ≤ 50% implied a lack of substantial heterogeneity, thereby supporting the use of a fixed effects model. A 95% confidence interval (CI) was calculated for each pooled estimate. Publication bias was assessed using funnel plots and Egger’s test via Stata 17.0.

## 3 Results

### 3.1 Literature search results

**Figure 1:**
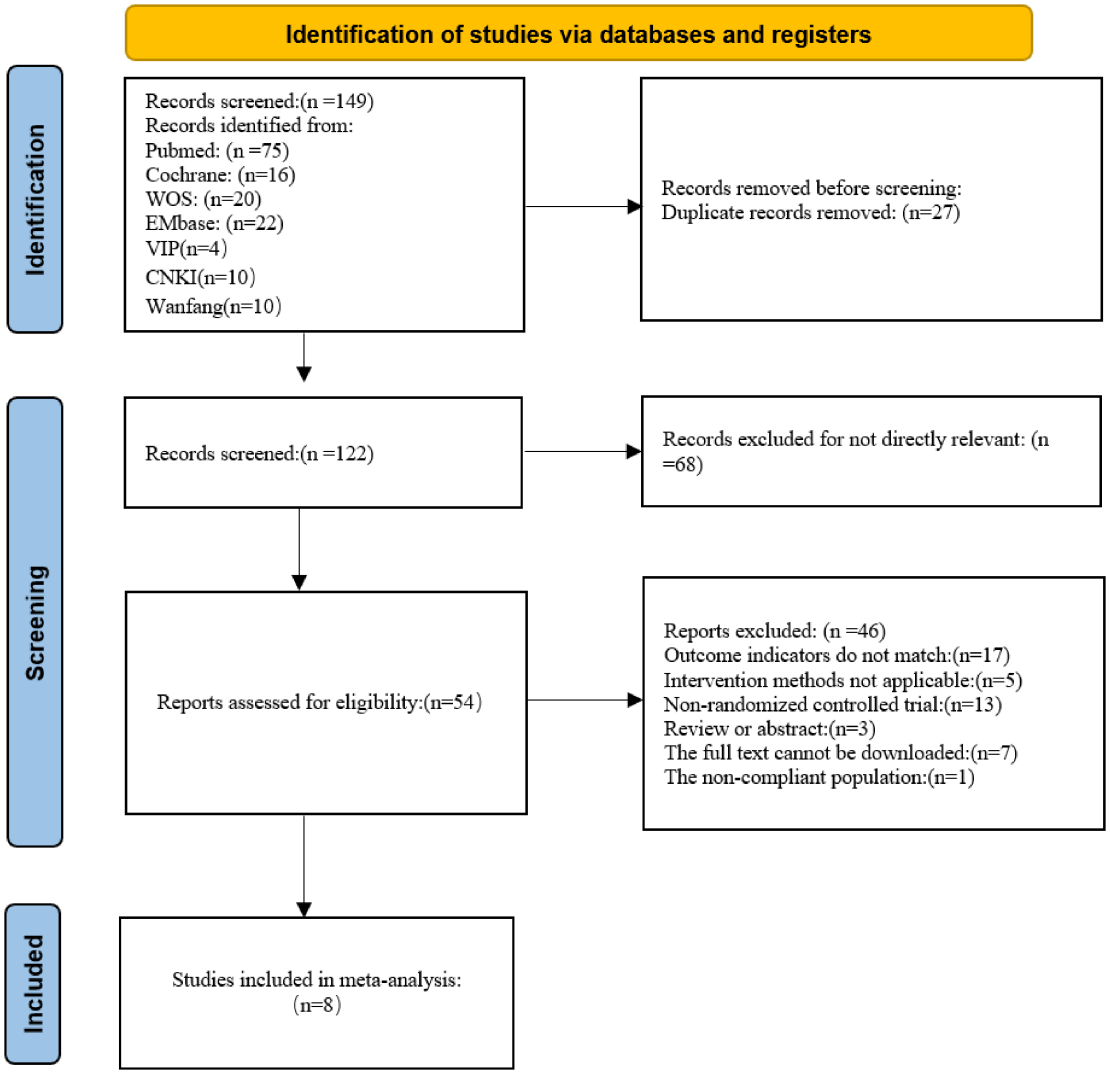
Literature screening process.

A total of 168 potentially relevant records were initially identified. After screening, eight RCTs (1, 12, 20–25) met the inclusion criteria and were included in the final analysis. The literature selection process is illustrated in Figure 1.

### 3.2 Study characteristics

The included studies involved 607 patients, all diagnosed with DEACMP. The intervention groups received NIBS for 20–30 days, with treatment administered 1–2 times per day. No adverse events related to NIBS were reported in any of the included studies. The detailed study characteristics are summarized in Table 3.

**Table 3:** Basic information of the included literature.

Study	Sample size	Sex (male/female)		Age (year)		Latency (d)		Intervention	Period (d)	Stimulus point	Assessment tools
	(T/C)	(T/C)		(T/C)		(T/C)		(T/C)			
Qi et al., 2022 (1)	39/41	–	–	60.5 ± 11.3	59.9 ± 12.5	15.5 ± 4.6	14.8 ± 4.9	A + B/A + C	28d	Anode: a Cathode: b	④⑤
Chen et al., 2023 (12)	60/64	39/25	34/26	44.9 ± 5.5	42.8 ± 3.6	22.4 ± 1.7	20.5 ± 3.3	D + B/D	20d	a	①②
Cao et al., 2021 (20)	20/20	9/11	12/8	59 ± 10	62 ± 8	23 ± 9	19 ± 7	A + D + B/A + D	30d	Anode: a Cathode: d	①②
Cao et al., 2020 (21)	40/40	12/14	13/12	51.52 ± 6.21	53.65 ± 6.95	22.8 ± 8.75	20.3 ± 7.22	A + B + D/A + C + D	30d	Anode: a Cathode: b	①②
Gong et al., 2017 (22)	34/30	19/15	17/13	41.5 ± 13.7	45.7 ± 11.9	–	–	A + B + D/A + D	20d	e	①②
Wang et al., 2017 (23)	22/20	14/8	13/7	47.05 ± 7.57	44.25 ± 9.79	11.0 ± 3.85	12.6 ± 4.75	A + B + D/A + D + C	20d	a	①②
Xin et al., 2024 (24)	41/41	24/17	20/21	48.26 ± 5.53	49.12 ± 5.48	–	–	A + B/A	28d	Anode: a Cathode: b	①
Zhang et al., 2024 (25)	58/59	27/30	32/28	18–78	18–78	18.3 ± 7.0	18.9 ± 8.0	A + D + B/A + D	10d	c	②

A: Conventional rehabilitation (including IV injection of drops). B: Electrical stimulation. C: Pseudo-electrical stimulation therapy (no current). D: Hyperbaric oxygen therapy. “–”: not reported.

a: Left dorsolateral prefrontal, b: Contralateral shoulder, c: Posterior to the root of the mastoid process bilaterally, d: Contralateral shoulder or right orbit, and e: Behind the ears at the mastoid process on both sides.

MMSE: Mini–Mental State Examination; ② BI: Barthel Index; ③ FIM: Functional Independence Measure; ④ MoCA: Montreal Cognitive Assessment Scale; ⑤ ADL: Ability to Perform Activities of Daily Living Scale.

### 3.3 Quality assessment of the included studies

All eight included studies were RCTs (1, 12, 20–25). They met the criteria for randomization, baseline comparability, intention-to-treat analysis, between-group statistical comparisons, and point estimates with variability measures. Only one study employed a blinded outcome assessment. The PEDro scores ranged from 6 to 8, with a mean score of 6.3, indicating overall good methodological quality. No studies were rated as low quality. The detailed quality scores are shown in Table 4.

**Table 4:** Methodological quality of the included studies.

	1^a^	2^b^	3^c^	4^d^	5^e^	6^f^	7^g^	8^h^	9^i^	10^j^	11^k^	Total score
Qi et al., 2022 (1)	1	1	0	1	0	0	0	1	1	1	1	6
Chen et al., 2023 (12)	1	0	0	1	0	0	1	1	1	1	1	6
Cao et al., 2021 (20)	1	1	0	1	0	0	0	1	1	1	1	6
Cao et al., 2020 (21)	1	1	0	1	0	0	0	1	1	1	1	6
Gong et al., 2017 (22)	1	1	0	1	0	0	0	1	1	1	1	6
Wang et al., 2017 (23)	1	1	0	1	0	0	0	1	1	1	1	6
Xing et al., 2024 (24)	1	1	0	1	0	0	0	1	1	1	1	6
Zhang et al., 2024 (25)	1	1	0	1	0	1	1	1	1	1	1	8

a Eligibility criteria.

b Random allocation.

c Assignment hiding.

d Baseline similarity.

e Blindness of the study population.

f Therapist blindness.

g Results-based assessment of blindness.

h Participation rate greater than 85%.

i Intention-to-treat analysis.

j Analysis of statistical results between groups.

k Point measurements and difference values.

### 3.4 Meta-analysis results

#### 3.4.1 Overall effects

##### 3.4.1.1 Effect of NIBS on cognitive function

A total of eight studies encompassing 607 participants compared the effects of NIBS with sham stimulation on cognitive function. As presented in Table 5, NIBS significantly enhanced cognitive function in patients with DEACMP [SMD = 1.03, 95% CI: 0.76 to 1.30; *p* < 0.001].

**Table 5:** Meta-analysis results of the effects of NIBS on cognitive function and ADL in patients with DEACMP.

Outcome indicator		Number of studies included	*I*^2^/%	Results of meta-analysis	
				SMD (95%CI)	*p*-value
Cognitive function		8 (607) (1, 12, 20–25)	95	1.03 (0.76, 1.3)	<0.00001
Age	<50 years old	4 (312) (12, 22–24)	0	1.22 (0.98, 1.46)	<0.00001
	≥50 years old	3 (178) (1, 22, 23)	79	0.80 (0.11, 1.49)	0.02
Incubation period	<20d	3 (239) (1, 23, 25)	68	0.87 (0.37, 1.37)	0.0006
	≥20d	3 (222) (12, 20, 21)	64	1.09 (0.58, 1.59)	<0.00001
Intervention duration	≤20d	4 (347) (12, 22, 23, 25)	0	1.09 (0.87, 1.32)	<0.00001
	20—30d	4 (260) (1, 20, 21, 24)	79	0.96 (0.37, 1.54)	0.001
Stimulation site	Left DLPFC	2 (166) (12, 23)	0	1.22 (0.89, 1.55)	0.66
	Yin and Yang	4 (260) (1, 20, 21, 24)	79	1.03 (0.76, 1.30)	0.002
	Other	2 (181) (22, 25)	0	0.99 (0.68, 1.29)	0.9
ADL		7 (525) (1, 12, 20–23, 25)	97	1.77 (0.43, 3.11)	<0.00001
Age	<50 years old	3 (230) (12, 22, 23)	98	1.54 (−0.63, 3.71)	0.16
	≥50 years old	3 (178) (1, 20, 21)	46	0.63 (0.21, 1.04)	0.003
Intervention duration	≤20d	4 (347) (12, 22, 23, 25)	98	2.68 (0.23, 5.13)	0.03
	20—30d	3 (178) (1, 20, 21)	46	0.63 (0.21, 1.04)	0.003
Stimulation site	Left DLPFC	2 (166) (12, 23)	98	2.07 (−1.36, 5.50)	0.24
	Yin and Yang	3 (178) (1, 20, 21)	46	0.63 (0.21, 1.04)	0.003
	Other	2 (181) (22, 25)	99	3.31 (−2.24, 8.86)	0.24

##### 3.4.1.2 Effect of NIBS on ADL

A total of seven studies involving 525 participants evaluated the effects of NIBS on ADL. The results indicated that NIBS significantly improved functional independence in patients with DEACMP [SMD = 1.77, 95% CI: 0.43 to 3.11; *p* < 0.001] (Table 5).

#### 3.4.2 Subgroup analyses

Subgroup analyses were conducted to explore sources of heterogeneity and to assess potential moderators of NIBS effectiveness, including age, latency, intervention duration, and stimulation site (Table 5).

##### 3.4.2.1 Cognitive function and daily living subgroups

For cognitive function, the subgroup analyses revealed no statistically significant effects when stimulation was applied to the left (DLPFC) or other non-bilateral sites (*p* > 0.05). However, all other subgroup comparisons yielded significant results (*p* < 0.05). For daily living outcomes, non-significant results were observed in patients aged <50 years and in those who received stimulation at the DLPFC or other non-bilateral sites. All other subgroup comparisons demonstrated statistically significant improvements.

From the perspective of heterogeneity sources, the heterogeneity of effects related to age, intervention duration, and stimulation site was consistently below 50%, demonstrating a substantial reduction in variance. These findings suggest that age, intervention duration, and stimulation site may serve as significant sources of heterogeneity in cognitive function. Concerning ADL, the heterogeneity of effects associated with age, intervention duration, and stimulation site revealed a notable decrease. Therefore, age, intervention duration, and stimulation site may similarly represent key sources of heterogeneity in daily living abilities.

#### 3.4.3 Sensitivity analysis

**Figure 2:**
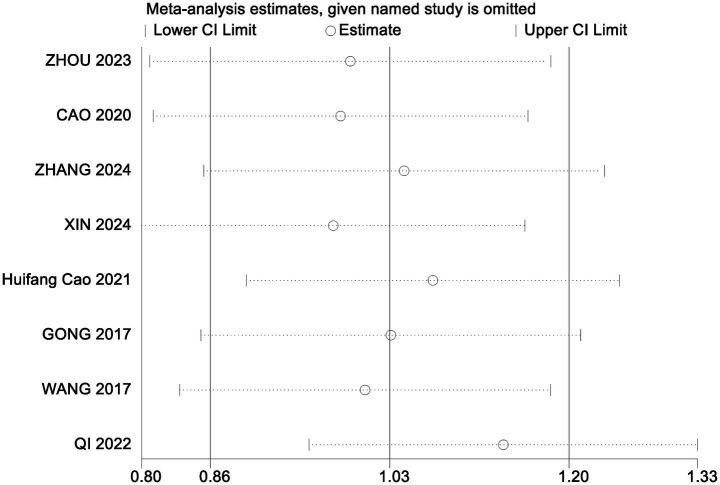
Sensitivity analysis of cognitive functioning.

**Figure 3:**
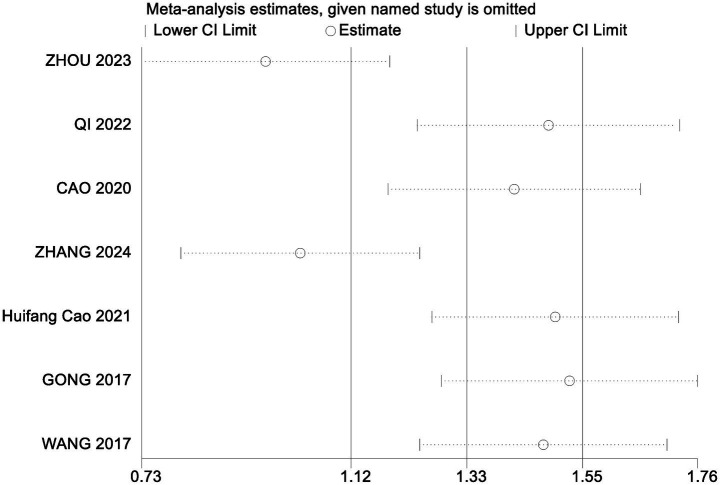
Sensitivity analysis of the ability to perform activities of daily living.

To assess the robustness of the meta-analysis findings and identify studies contributing to heterogeneity, a sensitivity analysis was performed by sequentially removing individual studies (Table 6). Excluding the study by Qi Hongna (1), which included older participants (mean age >60 years), resulted in a pooled effect size of SMD = 1.13 (95% CI: 0.91 to 1.35; *p* < 0.001), with heterogeneity reduced from 58 to 25%. This suggests that age may be a key source of variability. Across all sensitivity iterations, the pooled SMDs ranged from 0.97 to 1.09 and *I*^2^ ranged from 57 to 64%, with all *p*-values remaining <0.001, indicating stable and reliable results. Bubble diagrams are shown in Figures 2, 3.

**Table 6:** Combined effects of excluding individual studies on cognitive function and activities of daily living.

	Study	Effect size	95%CI	*P*-value	*I*^2^/%
Cognitive function	Qi 2022 (1)	1.13	0.91, 1.35	<0.001	25
	Chen 2023 (12)	1.01	0.69, 1.33	<0.001	62
	Cao 2021 (20)	1.09	0.81, 1.37	<0.001	57
	Cao 2020 (21)	0.97	0.7, 1.25	<0.001	56
	Gong 2017 (22)	1.04	0.73, 1.35	<0.001	64
	Wang 2017 (23)	1.00	0.71, 1.29	<0.001	62
	Xing 2024 (24)	0.98	0.69, 1.27	<0.001	57
	Zhang 2024 (25)	1.05	0.72, 1.37	<0.001	64
ADL	Qi 2022 (1)	1.94	0.31, 3.57	<0.001	98
	Zhou 2023 (12)	1.42	0.14, 2.7	<0.001	97
	Cao 2021 (20)	2.05	0.54, 3.55	<0.001	98
	Cao 2020 (21)	1.93	0.34, 3.52	<0.001	98
	Gong 2017 (22)	1.99	0.41, 3.57	<0.001	98
	Wang 2017 (23)	2.01	0.49, 3.54	<0.001	98
	Zhang 2024 (25)	1.06	0.05, 2.08	<0.001	95

For ADL, heterogeneity remained high across all sensitivity analyses, with *I*^2^ ranging from 95 to 98% and pooled SMDs from 1.06 to 2.05 (*p* < 0.001 for all comparisons). These findings suggest that while variability was present, the direction and significance of the effect remained robust.

### 3.5 Publication bias

**Figure 4:**
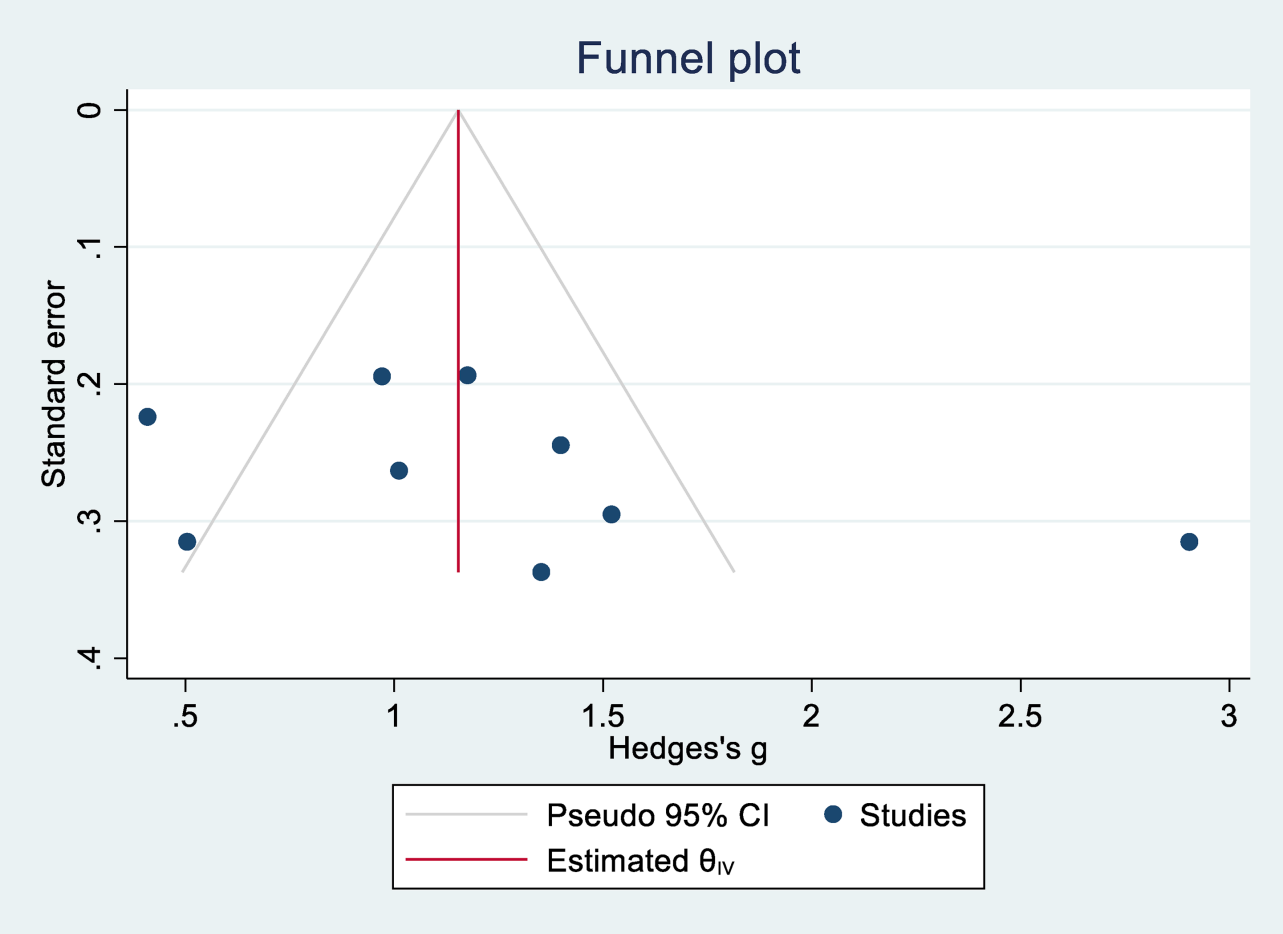
Cognitive publication bias.

**Figure 5:**
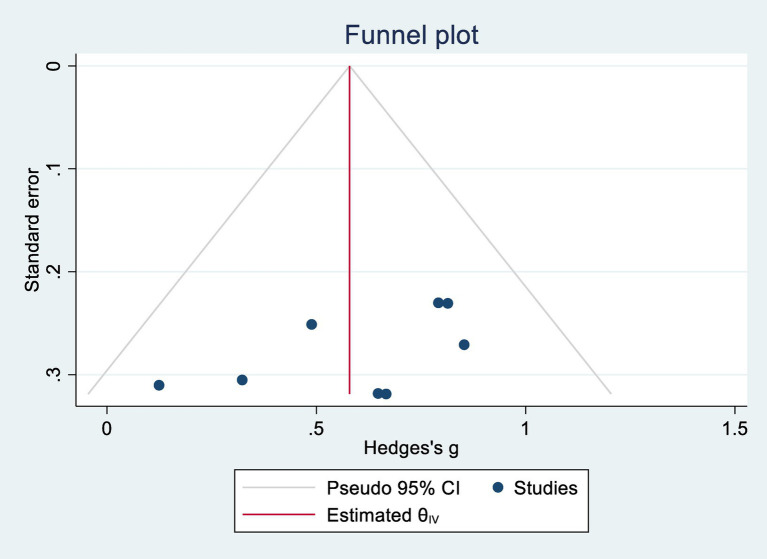
Daily life activity capacity publication bias.

Egger’s regression tests were conducted to assess potential publication bias. For cognitive function, *p* = 0.302; for ADL, *p* = 0.088. Both values exceeded the 0.05 threshold, indicating no significant publication bias. Corresponding funnel plots are shown in Figures 4, 5.

### 3.6 Quality of evidence

According to the GRADE assessment, the quality of evidence was rated as moderate for both cognitive function and ADL (Table 7).

**Table 7:** GRADE quality of evidence evaluation.

Outcome indicator	Inclusion of studies	Evaluation of the quality of evidence					Quality of evidence
		Research limitations	Inconsistency	Indirectness	Inaccuracy	Publication bias	
Cognitive function	8	A	B	B	B	B	Middle
ADL	7	A	B	B	B	B	Middle

A: serious; B: not serious.

### 3.7 Adverse events

Among the eight included studies (1, 12, 20–25), only two reported adverse events. In the study by Cao Shuangqing (21), four patients experienced mild erythema, itching, and tingling at the electrode site during stimulation, which resolved within 10 min after adjustment. Post-intervention, three patients reported transient symptoms including drowsiness, fatigue, headache, dizziness, and difficulty concentrating, all resolving spontaneously within 30–120 min. In the study by Xing Juan (24), several participants experienced adverse effects such as headache, liver dysfunction, and pruritus during treatment. No serious adverse events were reported in any study.

## 4 Discussion

This systematic review included eight randomized controlled trials assessing the efficacy of (NIBS) on cognitive function and (ADL) in patients with (DEACMP). The methodological quality of the included studies was generally high, with an average PEDro score of 6.3 and no low-quality trials identified. However, methodological limitations were observed: the majority of the studies did not adequately report allocation concealment or blinding procedures, which might have introduced subjective or informational bias. Only one study explicitly implemented both allocation concealment and assessor blinding. The meta-analysis demonstrated that the *I*^2^ values for both cognitive function and ADL exceeded 50%, reflecting considerable heterogeneity among the studies. The subgroup analysis further suggested that age, stimulation site, and intervention duration might constitute significant sources of this heterogeneity. Consequently, the quality of evidence concerning the effects of NIBS on cognitive function in patients with DEACMP was classified as moderate, with similar evidence quality for its effect on ADL.

Our findings demonstrate that NIBS significantly improves both cognitive function and ADL in patients with DEACMP. Notably, younger patients (<50 years) derived greater cognitive benefits, while older patients (≥50 years) exhibited more pronounced improvements in daily functioning. These outcomes suggest that age may serve as a key moderating factor. Furthermore, intervention durations of ≤20 days and stimulation sites located at bilateral anode–cathode poles were associated with greater therapeutic gains across both domains.

NIBS modalities such as transcranial direct current stimulation (tDCS) and repetitive transcranial magnetic stimulation (rTMS) exert neurophysiological effects by targeting specific cortical regions and modulating the excitability and connectivity of underlying neural networks (26, 27). These techniques have been particularly shown to enhance interconnectivity between the hippocampal and frontoparietal networks (28). tDCS induces long-term potentiation (LTP)-like plasticity by delivering a low-intensity direct current (1–2 mA) via scalp electrodes, whereas rTMS generates transient magnetic fields that depolarize neurons and induce cortical excitability changes (29). These techniques have been shown to enhance neuroplasticity and functional flexibility in individuals with mild cognitive impairment (MCI), thereby facilitating cognitive restoration (30–32). The subgroup analysis revealed that NIBS was particularly effective in patients under 50 years of age, with short intervention durations and stimulation targeting the bilateral poles. Conversely, older patients demonstrated greater ADL improvements under the same parameters. These age-related differences may be attributed to the natural decline in cerebrovascular function and neuroplasticity associated with aging. Aging is also accompanied by increased susceptibility to white matter demyelination, diminished neuronal repair capacity, and higher rates of delayed neurological recovery (33–36). These age-dependent mechanisms may account for the observed disparity in NIBS responsiveness between younger and older patients. The duration of intervention also emerged as a critical factor. Although our analysis suggested that shorter intervention periods (≤20 days) were associated with more substantial improvements, other studies have reported that multiple sessions (e.g., 5, 10, or 20 treatments) produce cumulative, dose-dependent effects on cognitive function (37). This discrepancy may be explained by neurorehabilitation theory, which posits that the brain enters a phase of heightened plasticity within the first month following injury, after which recovery rates taper off (38). The (DLPFC), particularly the left hemisphere, is implicated in executive, emotional, and attentional functions (21). Anodal stimulation of the left DLPFC has shown therapeutic benefits in depression (39) and has been associated with enhanced multitasking performance (40) and improved working memory (41). Stimulation configurations that use the left DLPFC as the anodal site and the right shoulder as the cathodal site may be optimal, as this montage forms a targeted current circuit while minimizing interference (42–44). In addition, cathodal stimulation of the left DLPFC has been associated with functional gains in ADL and motor recovery (45), underscoring its relevance as a neuromodulatory target in DEACMP rehabilitation.

Nevertheless, several limitations must be acknowledged. First, the included studies exclusively utilized tDCS and rTMS, limiting our ability to compare across different NIBS modalities. Second, only one study examined NIBS as a standalone intervention, while the remaining trials combined NIBS with hyperbaric oxygen therapy or conventional rehabilitation. This lack of isolated treatment arms precludes definitive conclusions regarding the sole efficacy of NIBS in this patient population. Similarly, due to the limited number of included studies and the lack of detailed categorization of cognitive functions, it is not possible to determine which specific cognitive domains are most improved by NIBS methods.

Despite these limitations, NIBS remains a promising, non-invasive, and well-tolerated intervention for neurorehabilitation (46). tDCS and rTMS—two of the most extensively studied NIBS techniques—modulate cortical function through central mechanisms and demonstrate a favorable safety profile. Although concerns about seizure risk have been raised, especially with high-frequency rTMS, low-frequency applications appear to have a more favorable safety margin (47, 48).

In summary, NIBS significantly enhances cognitive function and ADL in patients with DEACMP. Its effectiveness appears to be influenced by patient age, stimulation site, and intervention duration, with preliminary evidence suggesting a potential dose–response relationship. These findings provide valuable support for the clinical use of NIBS in DEACMP rehabilitation. However, as the current evidence base is limited, future studies with larger sample sizes, stratified NIBS modalities, and rigorous trial designs are warranted to establish precise treatment protocols.

## Data Availability

The original contributions presented in the study are included in the article/Supplementary material, further inquiries can be directed to the corresponding author.
