# Supplementary material for: Effect of non-invasive brain stimulation on cognitive function and activities of daily living in patients with carbon monoxide poisoning: a systematic review and meta-analysis
Source: Front Neurol. 2025 Aug 12;16:1585901. doi: 10.3389/fneur.2025.1585901 (PMC12379109; doi:10.3389/fneur.2025.1585901)
Supplement: Supplementary file 1 [file Data_Sheet_1.zip › Supplementary Materials/Text and Image Materials/Diagram in English.docx]

Figure 1 Literature screening process


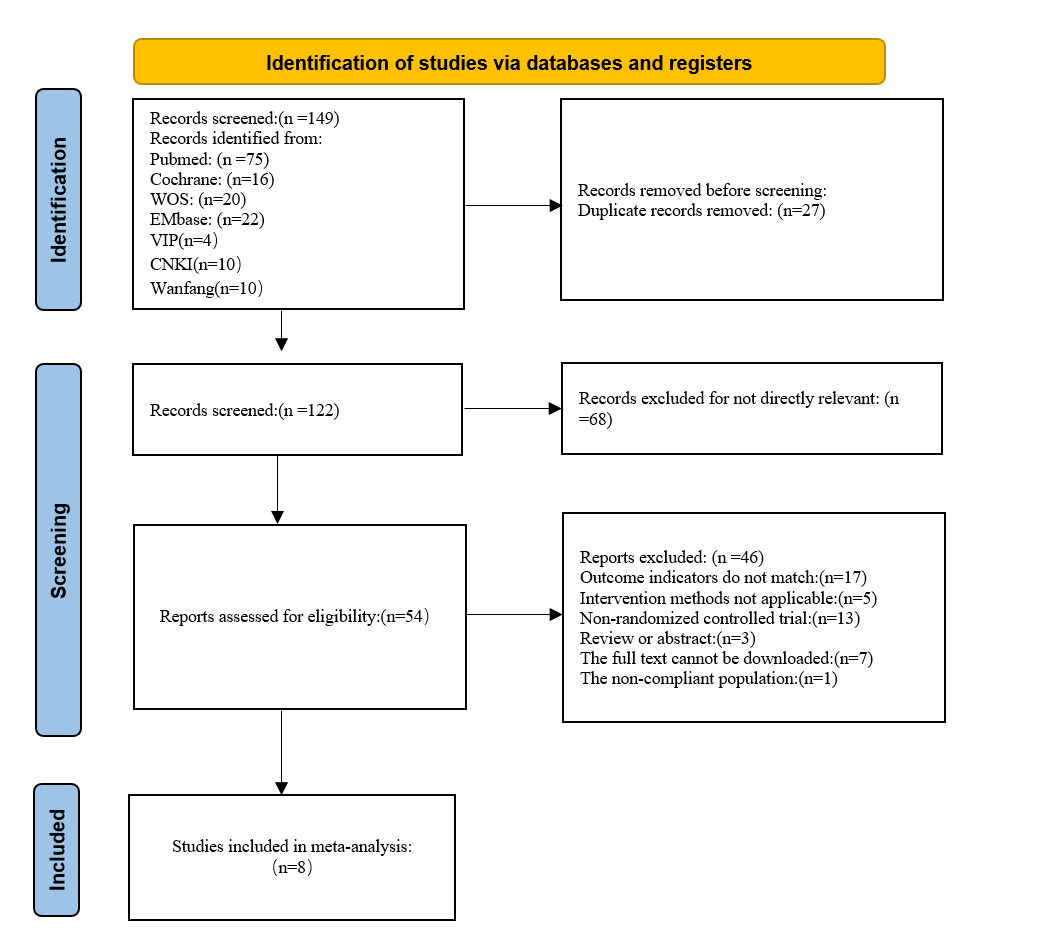


Figure 2 Sensitivity analysis of cognitive functioning

Figure 3 Sensitivity analysis of ability to perform activities of daily living

Figure 4 Cognitive publication bias

Figure 5 Daily life activity capacity publication bias
