# Supplementary material for: Effect of non-invasive brain stimulation on cognitive function and activities of daily living in patients with carbon monoxide poisoning: a systematic review and meta-analysis
Source: Front Neurol. 2025 Aug 12;16:1585901. doi: 10.3389/fneur.2025.1585901 (PMC12379109; doi:10.3389/fneur.2025.1585901)
Supplement: Supplementary file 1 [file Data_Sheet_1.zip › Supplementary Materials/Text and Image Materials/ú║Table in English .docx]

Table 1 PICOS architecture of non-invasive brain stimulation intervention for cognitive function in patients with delayed encephalopathy in carbon monoxide poisoning

| **Population** | **Intervention** | **Comparison** | **Outcome** | **Study Design** |
| --- | --- | --- | --- | --- |
| Patients with delayed encephalopathy after carbon monoxide poisoning (First episode, Age ≥ 18) | - Rehabilitation therapists - Hospital-based interventions - Brain stimulation techniques:    • Magnetic stimulation    • Electrical stimulation    • EEG-based synchronous bioelectric stimulation | 1. Brain stimulation group vs control group 2. Comparison of stimulation duration 3. Comparison of stimulation target points 4. Stratified analysis by age and disease latency | - Cognitive function - Activities of daily living (ADL) | Randomized Controlled Trial (RCT) |

Table 2 Literature search strategy

| comprehensive database | search step |
| --- | --- |
| PubMed与The Cochrane Library | #1“Carbon monoxide poisoning delayed encephalopathy”[Mesh] OR “carbon monoxide”[Title/Abstract] OR “Carbon monoxide poisoning” [Title/Abstract] OR “Illuminating Gas Poisoning” [Title/Abstract]  #2”Anodal Stimulation Transcranial Direct Current Stimulation” [Mesh] OR tDCS [Title/Abstract] OR ‘Transcranial direct current stimulation’ [Title/Abstract] OR ‘Transcranial alternating current stimulation’[Title/Abstract] OR 'repetitive transcranial magnetic stimulation' [Title/Abstract]  #3  “Cognitions”[Mesh]OR “Cognitive function” [Title/Abstract] OR “Cognitive Functions” [Title/Abstract]  #4 Randomized controlled trial [Publication Type] OR “Randomized” [Title/Abstract] OR “controlled” [Title/Abstract] OR “Trial” [Title/Abstract]  #5 #1 AND #2 AND #3 AND #4 |
| Web of Science | #1 TS=（“Carbon monoxide poisoning delayed encephalopathy”OR“carbon monoxide”OR“Carbon monoxide poisoning”OR“Illuminating Gas Poisoning”  #2 TS=（“Anodal Stimulation Transcranial Direct Current Stimulation” OR “Transcranial direct current stimulation” OR “repetitive transcranial magnetic stimulation”）  #3 TS=（“Cognitions” OR “Cognitive function” OR “Cognitive Functions”）  #4 TS=（“Randomized controlled trial” OR “Randomized” OR “Controlled” OR “Trial”）  #5 #1 AND #2 AND #3 AND #4 |
| Embase | #1 “Carbon monoxide poisoning delayed encephalopathy” [exp] OR “carbon monoxide” [ab,ti] OR “Carbon monoxide poisoning”[ab,ti] OR “Illuminating Gas Poisoning” [ab,ti]  #2 “Anodal Stimulation Transcranial Direct Current Stimulation” [exp] OR “Transcranial direct current stimulation” [ab,ti] OR “repetitive transcranial magnetic stimulation” [ab,ti]  #3 “Cognitions” [exp] OR “Cognitive function” [ab,ti] OR “Cognitive Functions”” [ab,ti]  #4 “Randomized controlled trial” [exp] OR “Randomized” [ab,ti] OR “Controlled” [ab,ti] OR “Trial” [ab,ti]  #5 #1 AND #2 AND #3 AND #4 |
| 中国知网 | 主题=（一氧化碳+一氧化碳中毒+一氧化碳中毒迟发性脑病） AND主题=（电刺激+经颅直流电刺激+经颅交流电刺激+磁刺激+非侵入脑刺激）AND主题=（认知功能+日常生活活动能力） |
| 万方、维普 | 主题=（一氧化碳OR一氧化碳中毒OR一氧化碳中毒迟发性脑病） AND主题=（电刺激OR经颅直流电刺激OR经颅交流电刺激0R磁刺激0R非侵入脑刺激）AND主题=（认知功能+日常生活活动能力） |

Table 3 Basic information of the included literature

| **study** | sample size | genders（males/ women） | | age（year） | | latency（d） | | intervention | period (d) | stimulus point | Assessment tools |
| --- | --- | --- | --- | --- | --- | --- | --- | --- | --- | --- | --- |
|  | （T/C) | （T/C) | | （T/C) | | （T/C) | | （T/C) |  |  |  |
| Zhou 2023^[24]^ | 60/64 | 39/25 | 34/26 | 44.9±5.5 | 42.8±3.6 | 22.4±1.7 | 20.5±3.3 | D+B/D | 20d | a | ①③ |
| Qi 2022^[1]^ | 39/41 | -- | ---- | 60.5±11.3 | 59.9±12.5 | 15.5±4.6 | 14.8±4.9 | A+B/A+C | 28d | Anode: a cathode:b | ④⑤ |
| Cao 2020^[19]^ | 40/40 | 12/14 | 13/12 | 51.52±6.21 | 53.65±6.95 | 22.8±8.75 | 20.3±7.22 | A+B+D/A+C+D | 30d | Anode:a cathode:b | ①② |
| Zhang 2024^[23]^ | 58/59 | 27/30 | 32/28 | 18-78 | 18-78 | 18.3±7.0 | 18.9±8.0 | A+D+B/A+D | 10d | c | ①② |
| Xin 2024^[22]^ | 41/41 | 24/17 | 20/21 | 48.26±5.53 | 49.12±5.48 | ------ | ----- | A+B/A | 28d | Anode: a cathode: b |  |
| Cao 2021^[18]^ | 20/20 | 9/11 | 12/8 | 59±10 | 62±8 | 23±9 | 19±7 | A+D+B/A+D | 30d | Anode: a cathode: d | ①② |
| Gong 2017^[20]^ | 34/30 | 19/15 | 17/13 | 41.5±13.7 | 45.7± 11.9 | ---- | ----- | A+B+D/A+D | 20d | e | ①② |
| Wang 2017^[21]^ | 22/20 | 14/8 | 13/7 | 47.05 ±7.57 | 44.25± 9.79 | 11.0±3.85 | 12.6±4.75 | A+B+D/A+D+C | 20d | a | ①② |

Note: A:Conventional rehabilitation (including IV injection of drops) B:Electrical stimulation C:Pseudo-electrical stimulation therapy (no current) D:Hyperbaric oxygen therapy “---”: not reported

a:Left dorsolateral prefrontal b: contralateral shoulder c: Posterior to the root of the mastoid process bilaterally d:contralateral shoulder or right orbit e:Behind the ears at the mastoid process on both sides

MMSE: Brief Mental Status Examination ② BI: Barthel Index ③ FIM: Functional Independence Measure ④ MOCA: Montreal Cognitive Assessment Scale ⑤ ADL: Ability to Perform Activities of Daily Living Scale

Table 4 Methodological quality of the included studies.

|  | 1^a^ | 2^b^ | 3^c^ | 4^d^ | 5^e^ | 6^f^ | 7^g^ | 8^h^ | 9^i^ | 10^j^ | 11^k^ | Total score |
| --- | --- | --- | --- | --- | --- | --- | --- | --- | --- | --- | --- | --- |
| Zhou et al, 2023 | 1 | 0 | 0 | 1 | 0 | 0 | 1 | 1 | 1 | 1 | 1 | 6 |
| Qi et al, 2022 | 1 | 1 | 0 | 1 | 0 | 0 | 0 | 1 | 1 | 1 | 1 | 6 |
| Cao et al, 2020 | 1 | 1 | 0 | 1 | 0 | 0 | 0 | 1 | 1 | 1 | 1 | 6 |
| Zhang et al, 2024 | 1 | 1 | 0 | 1 | 0 | 1 | 1 | 1 | 1 | 1 | 1 | 8 |
| Xing et al, 2024 | 1 | 1 | 0 | 1 | 0 | 0 | 0 | 1 | 1 | 1 | 1 | 6 |
| Cao et al, 2021 | 1 | 1 | 0 | 1 | 0 | 0 | 0 | 1 | 1 | 1 | 1 | 6 |
| Gong et al, 2017 | 1 | 1 | 0 | 1 | 0 | 0 | 0 | 1 | 1 | 1 | 1 | 6 |
| Wang et al, 2017 | 1 | 1 | 0 | 1 | 0 | 0 | 0 | 1 | 1 | 1 | 1 | 6 |

^a^ eligibility criteria.

^b^ random allocation.

^c^ Assignment hiding.

^d^ Baseline similarity.

^e^ Blindness of the study population.

^f^ Therapist blindness.

^g^ Results-based assessment of blindness.

^h^ Participation rate greater than 85%.

^i^ Intention-to-treat analysis.

^j^ Analysis of statistical results between groups.

^k^ Point measurements and difference values.

Table 5 Meta-analysis results of the effect of NIBS on cognitive function and activities of daily living in DEACMP patients

| outcome indicator | | Number of studies included | | I²/% | Results of meta-analysis | |
| --- | --- | --- | --- | --- | --- | --- |
|  |  |  |  |  | SMD (95%CI) | P-value |
| cognitive function | | | 8(607) ^[1, 18-24]^ | 95 | 1.03(0.76,1.3) | ＜0.00001 |
|  |  |  |  |  |  |  |
| age | ＜50 age | | 4 (312)^[20-22, 24]^ | 0 | 1.22(0.98,1.46) | ＜0.00001 |
|  | ≥50 age | | 3 (178)^[1, 20, 21]^ | 79 | 0.80(0.11,1.49) | 0.02 |
| incubation period | ＜20d | | 3(239)^[1, 21, 23]^ | 68 | 0.87(0.37,1.37) | 0.0006 |
|  | ≥20d | | 3(222)^[18, 19, 24]^ | 64 | 1.09 (0.58,1.59) | ＜0.00001 |
| intervention cycle | ≤20d | | 4(347) ^[20, 21, 23, 24]^ | 0 | 1.09 [0.87, 1.32] | ＜0.00001 |
|  | 20—30d | | 4(260) ^[1, 18, 19, 22]^ | 79 | 0.96 [0.37, 1.54] | 0.001 |
| Stimulation point | Left prefrontal parieto-superior region | | 2(166)^[21, 24]^ | 0 | 1.22 [0.89, 1.55] | 0.66 |
|  | Yin and Yang | | 4(260)^[1, 18, 19, 22]^ | 79 | 1.03 [0.76, 1.30] | 0.002 |
|  | other | | 2(181)^[20, 23]^ | 0 | 0.99 [0.68, 1.29] | 0.9 |
| Activities of daily living | | | 7(525)^[1, 18-21, 23, 24]^ | 97 | 1.77(0.43,3.11) | ＜0.00001 |
| age | ＜50age | | 3(230)^[20, 21, 24, 25]^ | 98 | 1.54(-0.63,3.71) | 0.16 |
|  | ≥50age | | 3 (178)^[1, 18, 19]^ | 46 | 0.63(0.21,1.04) | 0.003 |
| incubation period | ≤20d | | 4(347) ^[20, 21, 23, 24]^ | 98 | 2.68 [0.23, 5.13] | 0.03 |
|  | 20—30d | | 3(178) ^[1, 18, 19]^ | 46 | 0.63 [0.21, 1.04] | 0.003 |
| stimulation point | Left prefrontal parieto-superior region | | 2(166)^[21, 26]^ | 98 | 2.07 [-1.36, 5.50] | 0.24 |
|  | Yin and Yang | | 3(178)^[1, 18, 19]^ | 46 | 0.63 [0.21, 1.04] | 0.003 |
|  | other | | 2(181)^[20, 23]^ | 99 | 3.31 [-2.24, 8.86] | 0.24 |

Table 6 Combined effects of excluding individual studies of cognitive function and activities of daily living ability

|  | study | effect size | 95%CI | P | I²/% |
| --- | --- | --- | --- | --- | --- |
|  | Huifang Cao 2021^[18]^ | 1.09 | 0.81,1.37 | ＜0.001 | 57 |
|  | Zhou2023^[24]^ | 1.01 | 0.69,1.33 | ＜0.001 | 62 |
|  | Zhang2024^[23]^ | 1.05 | 0.72,1.37 | ＜0.001 | 64 |
| cognitive | Cao 2020^[19]^ | 0.97 | 0.7,1.25 | ＜0.001 | 56 |
| function | Wang 2017^[21]^ | 1.00 | 0.71,1.29 | ＜0.001 | 62 |
|  | Xing 2024^[22]^ | 0.98 | 0.69,1.27 | ＜0.001 | 57 |
|  | Qi2022^[1]^ | 1.13 | 0.91,1.35 | ＜0.001 | 25 |
|  | Gong2017^[20]^ | 1.04 | 0.73,1.35 | ＜0.001 | 64 |
|  | Huifang Cao 2021^[18]^ | 2.05 | 0.54,3.55 | ＜0.001 | 98 |
|  | Zhou2023^[24]^ | 1.42 | 0.14,2.7 | ＜0.001 | 97 |
| Activities | Zhang2024^[23]^ | 1.06 | 0.05,2.08 | ＜0.001 | 95 |
| of Daily | Cao 2020^[19]^ | 1.93 | 0.34,3.52 | ＜0.001 | 98 |
| Living | Wang 2017^[21]^ | 2.01 | 0.49,3.54 | ＜0.001 | 98 |
|  | Qi2022^[1]^ | 1.94 | 0.31,3.57 | ＜0.001 | 98 |
|  | Gong2017^[20]^ | 1.99 | 0.41,3.57 | ＜0.001 | 98 |

Table 7 GRADE Quality of Evidence Evaluation

| outcome indicator | Inclusion of studies | Evaluation of the quality of evidence | | | | | Quality of evidence |
| --- | --- | --- | --- | --- | --- | --- | --- |
|  |  | Research  limitations | inconsistency | indirectness | inaccuracy | publication bias |  |
| cognitive | 8 | A | B | B | B | B | middle |
| ADL | 7 | A | B | B | B | B | middle |

A: severity B: not serious
