# Supplementary figures and images for: Effect of non-invasive brain stimulation on cognitive function and activities of daily living in patients with carbon monoxide poisoning: a systematic review and meta-analysis
Source: Front Neurol. 2025 Aug 12;16:1585901. doi: 10.3389/fneur.2025.1585901 (PMC12379109; doi:10.3389/fneur.2025.1585901)

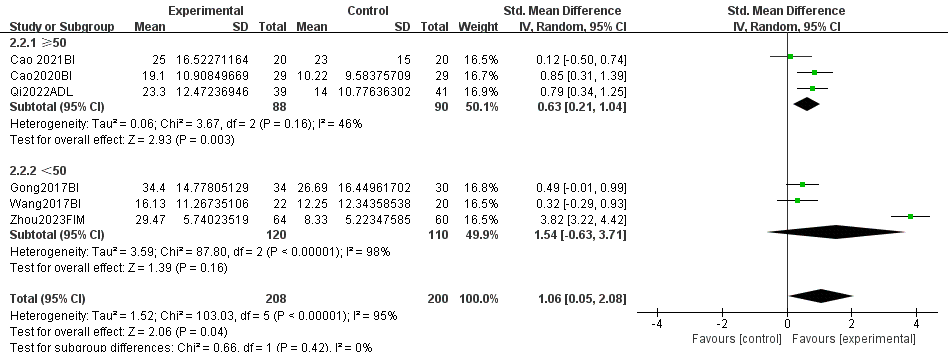

Supplement: Supplementary file 1 [file Data_Sheet_1.zip › Supplementary Materials/Subgroup Analysis/Age-based subgroup for Brain Injury (BI).png]

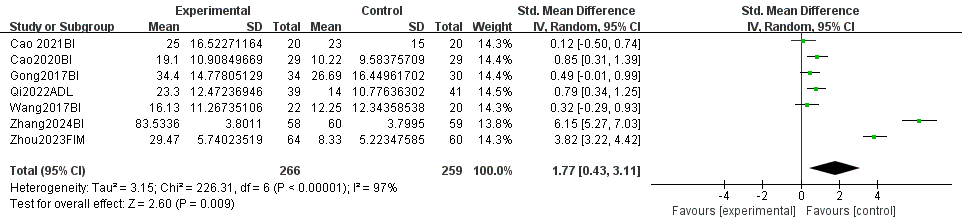

Supplement: Supplementary file 1 [file Data_Sheet_1.zip › Supplementary Materials/Subgroup Analysis/Forest plot for Brain Injury BI.png]

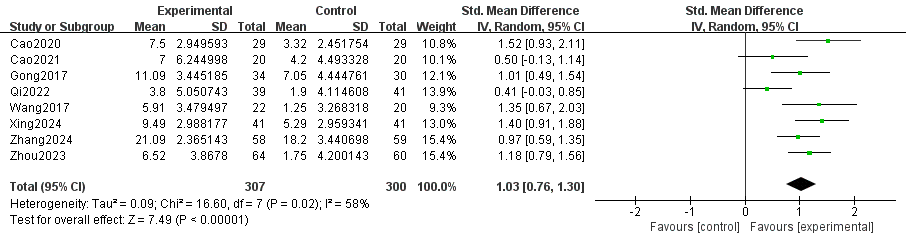

Supplement: Supplementary file 1 [file Data_Sheet_1.zip › Supplementary Materials/Subgroup Analysis/Forest plot for cognitive function.png]

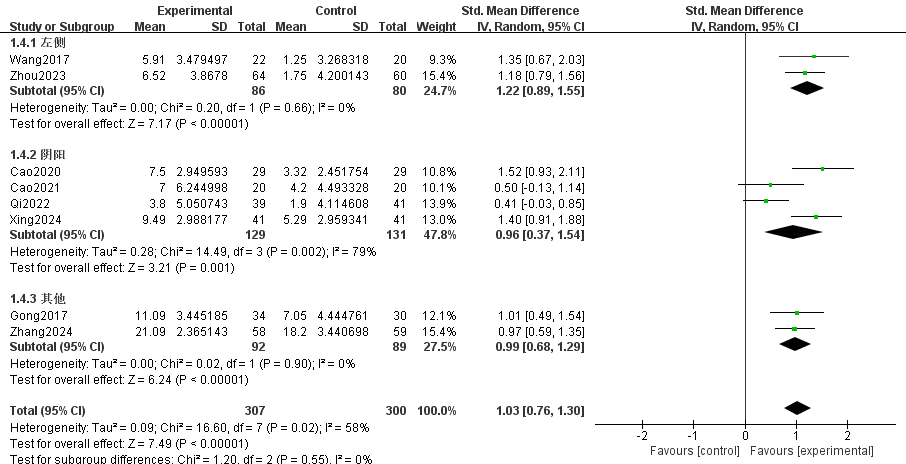

Supplement: Supplementary file 1 [file Data_Sheet_1.zip › Supplementary Materials/Subgroup Analysis/Stimulation site subgroups for BI.png]

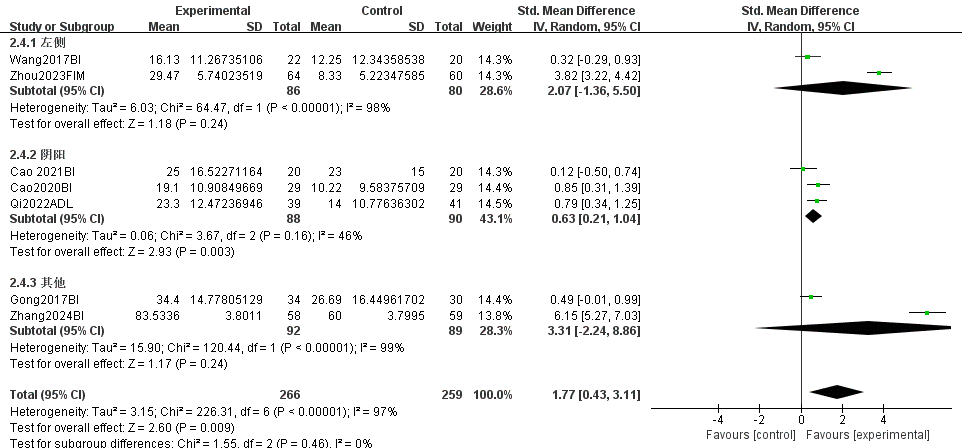

Supplement: Supplementary file 1 [file Data_Sheet_1.zip › Supplementary Materials/Subgroup Analysis/Stimulation site subgroups for cognitive function.png]

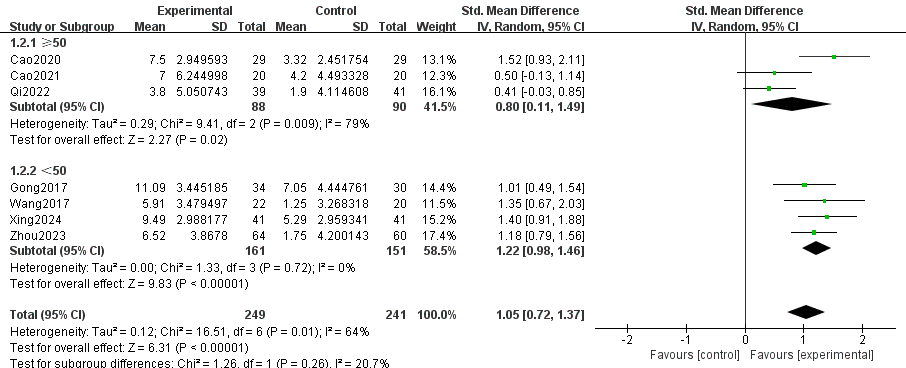

Supplement: Supplementary file 1 [file Data_Sheet_1.zip › Supplementary Materials/Subgroup Analysis/Subgroup based on age in cognitive function.png]

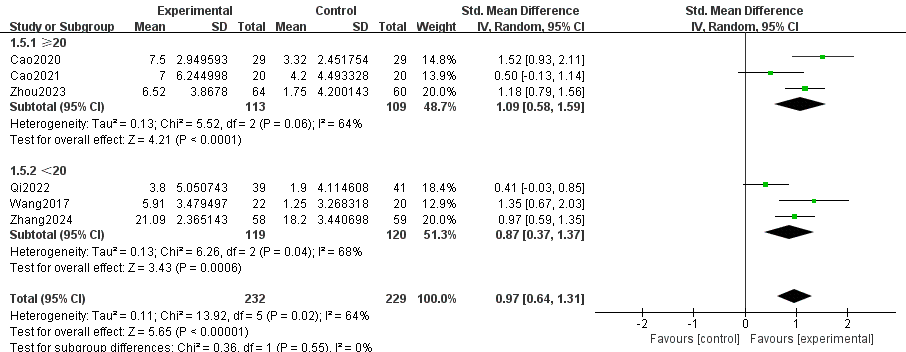

Supplement: Supplementary file 1 [file Data_Sheet_1.zip › Supplementary Materials/Subgroup Analysis/Subgroup based on latency period in cognitive function.png]

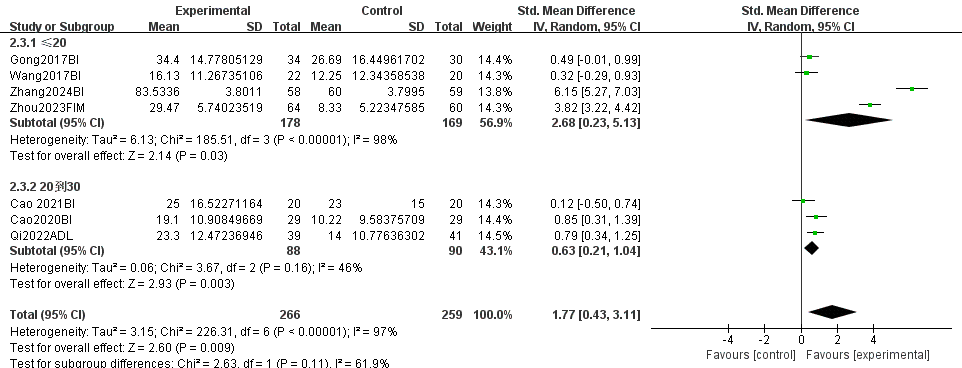

Supplement: Supplementary file 1 [file Data_Sheet_1.zip › Supplementary Materials/Subgroup Analysis/Subgroup Based on Temporal Patterns.png]

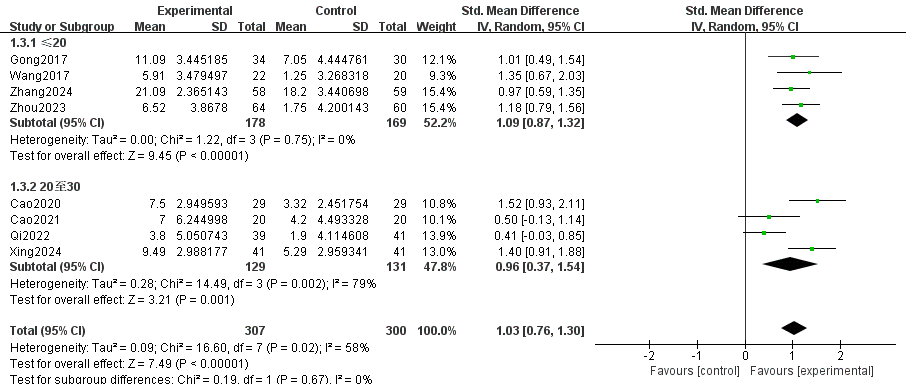

Supplement: Supplementary file 1 [file Data_Sheet_1.zip › Supplementary Materials/Subgroup Analysis/Subgroup of the Cognitive Cycle.png]
